# Autologous immuno magnetically selected CD133+ stem cells in the treatment of no-option critical limb ischemia: clinical and contrast enhanced ultrasound assessed results in eight patients

**DOI:** 10.1186/s12967-015-0697-4

**Published:** 2015-11-03

**Authors:** Vittorio Arici, Cesare Perotti, Calliada Fabrizio, Claudia Del Fante, Franco Ragni, Francesco Alessandrino, Gianluca Viarengo, Michele Pagani, Alessia Moia, Carmine Tinelli, Antonio Bozzani

**Affiliations:** Vascular Surgery Unit, Fondazione IRCCS Policlinico S. Matteo and University of Pavia, Piazzale Golgi 19, 27100 Pavia, Italy; Haemotransfusional Service, Fondazione IRCCS Policlinico S. Matteo and University of Pavia, Pavia, Italy; Radiology Service, Fondazione IRCCS Policlinico S. Matteo and University of Pavia, Pavia, Italy; Anesthesiology and Intensive Care Unit 2, Fondazione IRCCS Policlinico S. Matteo and University of Pavia, Pavia, Italy; Statistics and Epidemiology Service, Fondazione IRCCS Policlinico S. Matteo and University of Pavia, Pavia, Italy

**Keywords:** Peripheral arterial disease, Critical limb ischemia, Stem cell therapy, Contrast enhanced ultrasound

## Abstract

**Objectives:**

Demonstrate the safety and effectiveness of highly purified CD133+ autologous stem cells in critical limb ischemia (CLI).

**Design:**

Prospective single-center not randomized. Clinicaltrials.gov identifier: NCT01595776

**Methods:**

Eight patients with a history of stable CLI were enrolled in a period of 2 years. After bone marrow stimulation and single leukapheresis collection, CD133+ immunomagnetic cell selection was performed. CD133+ cells in buffer phosphate suspension was administered intramuscularly. Muscular and arterial contrast enhanced ultra sound (CEUS), lesion evolution and pain management were assessed preoperatively and 3, 6 and 12 months after the implant.

**Results:**

No patient had early or late complications related to the procedure. Two patients (25 %) didn’t get any relief from the treatment and underwent major amputation. Six patients (75 %) had a complete healing of the wounds, rest pain cessation and walking recovery. An increase in CEUS values was shown in all eight patients at 6 months and in the six clinical healed patients at 12 months and had statistical relevance.

**Conclusions:**

Highly purified autologous CD133+ cells can stimulate neo-angiogenesis, as based on clinical and CEUS data.

**Electronic supplementary material:**

The online version of this article (doi:10.1186/s12967-015-0697-4) contains supplementary material, which is available to authorized users.

## Background

Symptomatic peripheral arterial disease (PAD) has a prevalence of 3 % in a population aged 40 years and above and of 6 % in patients over 60. Critical Limb Ischemia (CLI) is the worst and terminal clinical picture of PAD often preceding gangrene and amputation: typical symptoms are rest pain refractory to analgesics lasting more than 2 weeks and ischemic lesions (Fontaine classification stage 3–4 and Rutherford classification stage 4–6). CLI diagnosis is confirmed instrumentally by calf arterial pressure <50 mmHg, Ankle/Brachial Index (ABI) <0.5 and Transcutaneous PO_2_ (TcPO_2_) <30 mmHg. The Inter-Society Consensus for the Management of Peripheral Arterial Disease (TASC II) [1] stresses the absolute indication to revascularization for these patients either by surgical or endovascular treatment. Nevertheless in some cases, mostly due to lack of distal run off, revascularization is not feasible or shows very low success rates. For the above mentioned reasons, the prognosis of these CLI patients is poor, with a major amputation rate of about 50 % at 1 year.

Many non-interventional treatments have been proposed for these “no-options” patients: spinal cord stimulation, prostanoids and prostaglandins administration, hyperbaric oxygen therapy and so on. More recently some authors have focused the attention on the local administration of stem cells, and particularly Endothelial Progenitor Cells (EPCs). EPCs local administration effectiveness in post-ischemic myocardial damage has been demonstrated in animal models and in humans [2–5]. Many observations lead to argue that the EPCs play a basic role in re-endothelization and neo-vascularization processes: actually the EPCs number may be reduced in peripheral blood in cardiovascular diseases, diabetes and rheumatoid arthritis and under the action of exogenous (as smoke habit) or endogenous (as high C-reactive protein levels) factors. Otherwise EPCs number may be increased by factors as physical exercise, estrogens, erythropoietin, statins and several cytokines or secreting proangiogenic factors like hepatic growth factor (HGF), insulin like growth factor (IGF-1) and the vascular endothelial growth factor (VEGF). These observations persuaded some authors to apply this cell therapy approach to CLI [6].

Our starting experience in the treatment of no options CLI with EPCs in three patients has been previously reported [7]. The bench marks of our study are the use of a selected EPCs population to investigate more precisely the cellular mechanisms, and to assess whether patients with PAD treated with EPCs show variation in muscle perfusion as displayed by contrast enhanced ultrasound (CEUS).

According to the current guidelines, reliable diagnostic tools for the management of patients with PAD, are ankle-brachial index (ABI), duplex ultrasound, transcutaneous oxygen pressure (TcPO_2_), magnetic resonance (MR), contrast computer tomography (CT scan) and conventional angiography (CA). Among imaging modalities under development, micro bubble—based CEUS is a real-time, high spatial-resolution imaging technique that can easily be applied. Its high sensitivity, which provides detection of extremely low concentrations of micro bubbles, combined with the true blood pool distribution of contrast agent, gives CEUS the potential to help visualize and quantify the vasculature in vivo. CEUS was recently proposed as a valuable method to detect perfusion deficit and collateralization in patients with PAD [8, 9]. This method has been also validated for the detection of impaired microcirculation in patients with diabetes mellitus [10].

We present our experience in eight no-options CLI patients transplanted with peripheral immune-selected apheresis-derived autologous CD133+ cells and followed up with CEUS for 12 months.

## Patients and methods

### Type of the study

Prospective single centre not randomized.

*Clinicaltrials.gov* identifier: NCT01595776

### Aim of the study

To assess the safety, feasibility and efficacy of local intramuscular administration of autologous immuno-selected CD133+ cells in patients suffering from CLI. The protocol started at our institution in July 2011 with the local Ethical Committee (EC) approval (number CHVAS-01-08-10/03/08). The investigation conforms with the principles outlined in the Declaration of Helsinki.

### Enrolment criteria

All the patients enrolled were suffering from clinical stable CLI according to the TASC 2 definitions [1] and had no revascularization option, on the basis of contrast CT scan, RM or angiography imaging and the evaluation of our vascular and endovascular team. A detailed informed consent, approved by our EC, had been required.

### Exclusion criteria

Patients under 18 and over 70 years of age. Elderly patients have been excluded because of expected bone marrow low responsiveness. Clinical unsteadiness of CLI (such as gangrene requiring major amputation) and poor life expectancy are exclusion criteria for supposed latency of the EPCs action. Severe systemic illness was judged to increase the risk of bone marrow stimulation. Complete inclusion and exclusion criteria are depicted in Table 1.

**Table 1:** Inclusion and exclusion criteria

Inclusion criteria
Age between 18 and 70 years
CLI (TASC 2)
None surgical nor endovascular option
Written informed consent

Exclusion criteria
Clinical instability
Extensive gangrene (proximal to forefoot)
All serious systemic disease
Life expectancy <24 months
Child bearing age
Allergy
Previous similar studies
Previous experimental drug studies within 3 months
Conflict of interest with the study

### Patients

Between September 2011 and September 2013 we enrolled eight patients with a history of Rutherford stage 4 (rest pain) or 5 (small ischemic lesions) PAD. All patients had previous vascular imaging (contrast CT or MR or angiography) excluding revascularization options, either endovascular and surgical and encountered inclusion criteria. Every patient underwent routine physical and instrumental examination including electrocardiogram, chest X-ray and blood sample analysis. The patients’ features are summarized in Table 2. The median age was 46.8 (SD 11.8, range 37–69 years), with a M:F ratio of 3:1. Younger patients met Buerger disease criteria (n = 4, 50 %), whereas others had pure atherosclerotic lesions. Only one patient (ID 7, female) had diabetes mellitus. Six patients out of eight had ischemic lesions on the forefoot (Rutherford stage 5) with poor healing and a long history of wound treatment. All patients complained moderate/severe pain and took high doses of analgesics (slow release opiates in two cases).

**Table 2:** Patients baseline

ID	Sex	Age	Risk factors	Diagnosis	Lesion’ site and extension	Rest pain
1	m	57	AH, hyperuricemia, HC, ESH	PAD	Left leg ulcer, 16 cm	+++
2	m	39	ESH	BD	Left foot ulcer, 2 cm	++
3	m	37	Hyperhomocysteinemia, ESH	BD	Left forefoot amputation dehiscence, 15 cm	+++
4	m	69	AH, ESH, HC	PAD	Right foot ulcer, 5 cm	++
5	f	41	ESH	BD	Right foot ulcer, 1 cm	++
6	m	34	ESH	BD	Left finger amputation dehiscence, 10 cm	+++
7	f	52	AS, AH, DM	PAD	None	+++
8	m	49	ESH	PAD	None	++

*AH* arterial hypertension, *HC* hypercholesterolemia, *ESH* ex smoke habit, *PAD* peripheral arterial disease, *BD* buerger disease, *AS* active smoker, *DM* diabetes mellitus

Pain scale: +mild, ++ moderate, +++ severe

### CEUS imaging protocol

Two operators who were blinded to treatment performed CEUS for all patients. The imaging criteria were: (1) reduced transmit power, at approximately 7–10 frames per second and one focus well below the level of the target to ensure a more uniform pressure field. (2) dual-mode presentation of a grayscale image side-by-side with the contrast image facilitating real-time identification of anatomic structures and region of interest (ROI) selection. (3) Image loops of approximately 60 s. (4) Uniform gain across the image and avoid gain saturation. (5) The time gain compensation (TGC) set such that before contrast arrival a uniform black image was shown.

A vial of contrast agent (SonoVue BR1; Bracco, Milan, Italy) was prepared at a concentration of about 2 × 10^8^ sulfur hexafluoride—filled micro bubbles per milliliter, according to the manufacturer’s recommendations. The position of the probe was recorded for each patient in order to maintain the same position during follow up. The injection was made with the patient supine and after 10 min of rest to avoid exercise related micro-vascular dilatation. The radiologist maintained a constant image plane with the aid of the tissue (fundamental image) of the “Contrast Side/Side” imaging mode.

### Image analysis

The main image analysis tasks were: (1) identification of anterior tibialis artery (ATA) area, (2) selection of a representative region of normal anterior tibialis muscle (ATM) and (3) formulation of time-intensity curves (TIC). Two manually defined ROI, 2 and 4 cm sided-squares, were placed, respectively, over the ATM with no evidence of arterial branches, over ATA and over a small tibialis arterial branch. The ROIs were placed in the same anatomical position for each patient to avoid unwanted differences during follow up examinations. One TIC was obtained for each ROI. The image loops were transferred to a personal computer for further analysis. From the analysis of TIC, we computed regional blood flow (RBF) and regional blood volume (RBV). TIC were extracted using commercial quantification software (QontraXt v.3.60, AMID, Rome, Italy). This software allows manual ROI selection, measurement of the selected ROI area and provides linear data for the TIC. For the ROI in the normal ATM, effort was made to place the region in an area without large vessels. The ATA ROI was a 2 cm square area and the ATM ROI was a 4 cm square area. TICs were obtained by computing the mean intensity of pixels comprised within the ROI at each time point. For each image loop were calculated:

- RBV which consists in the total amount of contrast media within the selected ROI, in a period of time. Due to the characteristics of US contrast media, it reflects the quantity of blood in a defined region. It is directly related to the area under the curve (AUC).
- RBF consists in the contrast media flow (related to the blood flow) in a selected ROI. It is related to the mean transit time.

### Bone marrow stimulation

Human recombinant granulocyte colony-stimulating factor (rhGCSF) was administered subcutaneously for 4–5 consecutive days at a dosage of 10 µg/kg daily, split in two doses. Starting from the third day of stem cells mobilization, the CD34+/133+ cells count was monitored daily by cytofluorimetric analysis. The minimum CD34+/133+ cells count acceptable for leukapheresis collection was 20 and 10/µl, respectively. Patients were monitored for any G-CSF related side effects.

### Leukapheresis (LKF) collection

A single LKF collection was planned for each patient using a third generation cell separator device (Spectra Cobe, Lakewood, CO, USA), processing at least 2.5 blood volumes according to our internal protocol for stem cell collection. Immediately after the LKF collection, a sample from patient’s peripheral blood was taken for haemocytometric analysis to evaluate platelet count and haemoglobin levels. Each LKF collection was diluted with 10 % acid citrate dextrose (ACD-A) and maintained overnight at 4 °C degrees before immuno-magnetic cell selection.

### Immunomagnetic cell selection

CD133+ immunomagnetic cell selection (ICS) was performed the day after LKF collection using the Clini-MACS (Miltenyi Biotec) device according to the manufacturer’s standard protocol.

### Quality controls

A sample taken from the CD133 cell positive fraction was seeded for short term (14 days) clonogenic assays to evaluate the quality of immunoselected stem cells in terms of proliferative capacity. A standard mixture of methylcellulose plus recombinant human growth factors was employed (Stem Cell Technologies, Vancouver, BC, Canada; MACS Media, Miltenyi Biotec GmbH, Bergisch Gladbach,Germany). Microbial cultures on the waste bag containing the negative fraction were carried out to detect aerobic-anaerobic bacteria and fungal contamination. A sample of 10 ml was inoculated in the culture medium (Bact/Alert FA and BacT/Alert FN, Organon Teknika Corp., Durham, NC) and incubated for 10 days at 37 °C.

### Cytofluorimetric analysis

Samples obtained from peripheral blood before mobilization with G-CSF, at time of LKF and after immunomagnetic cell selection were analyzed by flow cytometry to evaluate the expression of specific stem cell and endothelial antigens. Becton–Dickinson FACSCanto was employed for all flow cytometric analysis with a lyse no-wash technique, using the following monoclonal antibodies: anti-CD45 fluorescein isothiocyanate (FITC) (Becton–Dickinson, San Jose, CA, USA), anti-CD34 Peridinin-chlorophyll-protein complex (PerCP) (8G12 clone, Becton–Dickinson), anti-CD133 phycoerythrin (PE) (AC133 clone, Miltenyi Biotec) and anti-VEGF-R2 allophycocyanin (APC) (R&D systems), following the manufacturer instructions.

Each sample was acquired with BD FACSCanto recording 100.000 events inside the lymphocyte plus monocyte gate. Data files were analyzed with FACS Diva 6.1 software. Viability was assessed using 7-amino-actinomycin D (7-AAD) (Molecular Probes, Eugene, OR, USA).

### Implant procedure

After loco-regional anesthesia and below the knee cutaneous disinfection, 45–48 ml of autologous CD133+ cell in buffer phosphate (Miltenyi Biotec) suspension was administered intramuscularly with 1 ml deep injections through a 18G needle. The injections were so allocated: 10 ml in the anterior compartment of leg, 10 ml in the superficial posterior compartment, 10 ml in the deep posterior compartment, 10 ml in the lateral compartment and the remaining part in the foot (Additional file 1: Figure S1).

### Baseline assessment and follow up

Pain assessment was carried out with a personal scale of 3 degrees (mild, moderate and severe) and the pain killing drugs use monitored. Ischemic lesions were treated weekly by a wound management skilled nurse. CEUS, lesion evolution and pain management were assessed at baseline and 3, 6 and 12 months after the implant.

### Statistical analysis

All quantitative variables were normally distributed (Shapiro–Wilk test) and so the results were expressed as mean values and standard deviation (SD); qualitative variables were summarized as counts and percentages. Pearson’s r coefficient was used to test correlation between two study variables. Linear regression models for repeated measure were used to assess the increase over time of the CEUS parameters. Data analysis was performed with STATA statistical package (release 11.1, 2010, Stata Corporation, College Station, TX, USA).

## Results

### Patient’s mobilization and LKF

No relevant side effects related to G-CSF administration were registered. A single LKF collection per patient was performed. No side effects were registered. Patients did not require any red blood cells or platelet transfusion after LKF procedures. Total nucleated cells (TNC) content and viability in peripheral blood of the eight patients enrolled are depicted in Table 3.

**Table 3:** Cytofluorimetric analysis

ID	PB at collection			LKF		IS	Recovery (%)	Purity (%)	CD133+ infused (10^6^/limb kg)
	WBC (10^3^/uL)	CD34+ (n/uL)	CD133+/CD34+ (n/uL)	CD34+ (10^6^)	CD133+/CD34+ (10^6^)	CD133+ (10^6^)			
1	48.6	51.0	34.5	311.4	232.3	140.6	60.5	92.5	30.0
2	47.2	95.9	56.6	486.0	484.7	233.1	48.1	89.2	41.1
3	52.9	105.7	105.5	737.9	706.3	361.1	51.1	93.9	42.2
4	39.1	35.2	25.1	401.0	282.6	100.3	35.5	92.9	16.4
5	51.6	63.1	50.7	333.0	205.4	20.4	9.9	37.2	2.6
6	77.0	55.4	42.2	369.2	317.6	94.0	29.6	90.0	16.1
7	41.7	47.7	7.0	223.0	154.9	126.5	81.7	96.5	20.1
8	26.6	58.5	44.1	603.5	455.2	238.9	52.5	91.1	33.2
MV	48.1	54.7	45.7	433.1	354.9	164.4	46.1	85.4	25.2

*PB* peripheral blood, *LKF* leukapheresis, *IS* immunoselection, *WBC* white blood cells, *MV* mean values

### Immunomagnetic cell selection

The immunomagnetic cell selection was carried out the day after LKF collection. Almost all cells expressing CD133 antigen also expressed CD34 antigen. The mean CD133+ cell recovery was 46.1 % (SD 21.5, range 9.9–81.7). The mean purity was 85.4 % (SD 19.6, range, 37.2–96.5). TNC viability was always >90 %. Results of cytofluorimetric analysis performed on samples obtained from the positive fraction are detailed in Table 3. The mean number of CD133+ infused cell per limb kilogram was 25.2 × 10^6^ (SD 13.8; range 2.6–42.2). Clonogenic assays demonstrated the maintained proliferative capacity of immunoselected stem cells. The result of microbial cultures was always negative.

### Clinical results

No patient had early or late complications related to the procedure. Two patients (number 3 and 7, 25 %) didn’t get any relief from the treatment and underwent major amputation. Patient number 3 had lesion and pain worsening and was amputated below the knee after 5 months. Patient number 7 had pain worsening and final gangrene of the foot and underwent above the knee amputation after 7 months; this patient had diabetes and a heavy smoking habit (40 cigarettes/day) persisting in spite of physician’s indication. Five patients (number 1, 2, 4, 5 and 6, 62.5 %) had a complete healing of the wounds, complete rest pain cessation and walking recovery or increased pain free walking distance. One patient (number 8, 12.5 %) had rest pain cessation, and a mild improvement in pain free walking distance. None statistical correlation has been found between the number of infused CD133+ cells and the clinical results.

### CEUS

**Fig. 1:**
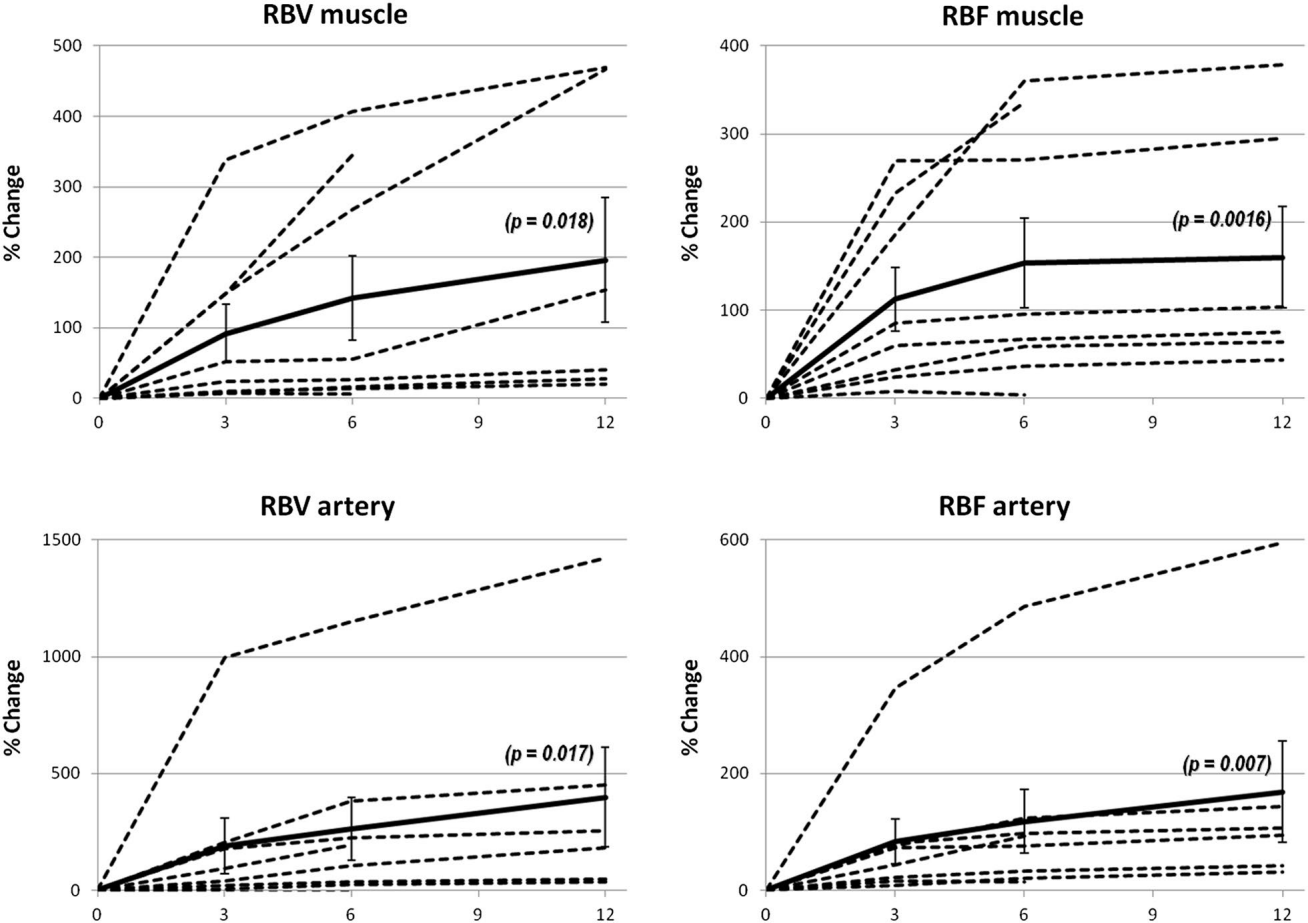
The diagrams show the percentage increase of both RBV and RBF both in muscle and in anterior tibial artery. *RBF* regional blood flow, *RBV* regional blood volume. In the *x axis* are months of follow-up. In the *y-axis* is the percentage of increase of the values of RBV and RBF compared to the baseline. *Dotted lines* represent single patient values. *Continue line* represents the mean percentage increase with the statistical significance. P values compare the value of mean increase to the baseline

An increase in RBF and RBV was shown in all eight patients at 3 and 6 months and in the six clinical healed patients at 12 months and has statistical relevance: at 12 months mean increasing for TAM-RBV was 48.8 % (*p* = 0.018), for TAM-RBF 59.4 % (*p* = 0.0016), for ATA-RBV 52.8 % (*p* = 0.017) and for ATA-RBF 48.6 (*p* = 0.007). The trends in increasing values at 3, 6 and 12 months for RBV and RBF, both in artery and in muscle, are depicted in Fig. 1. No statistical correlation between RBF and RBV value, and CD133+/CD34+ infused cells was found at 6 and at 12 months. Additional file 2: Figure S2 shows the correlation between clinical healing and CEUS values improvement in patient 1. Table 4 shows a synopsis of the results.

**Table 4:** Results

ID	Follow-up (months)	Early and late procedure related complications	Lesions healing	Limb salvage	Pain control^a^	CEUS assessment											
						TAM-RBV			TAM-RBF			ATA-RBV			ATA-RBF		
						Baseline	12 months	Δ %	Baseline	12 months	Δ %	Baseline	12 months	Δ %	Baseline	12 months	Δ %
1	12	None	Complete	Yes	+++	547.17	1391.05	+60.7	21.3	43.35	+50.8	661.37	3645.24	+81.8	29.61	72.08	+58.9
2	12	None	Complete	Yes	+++	40.89	231.72	+82.4	3.15	12.43	+74.6	620.4	2199.88	+71.8	45.89	89.03	+48.4
3^b^	5	None	None	No	+	358.13	(382.13)	(+6.2)	(15.03)	(15.6)	(+3.6)	1111.76	983.62	−13.0	41.73	47.86	+12.8
4	12	None	Complete	Yes	+++	1486.92	1789.42	+16.9	42.42	69.48	+38.9	1391.05	3899.2	+64.3	43.35	89.89	+51.8
5	12	None	Complete	Yes	+++	629.66	881.69	+28.6	21.68	38.00	+42.9	1468.95	1978.16	+25.7	50.87	66.58	+23.6
6	12	None	Complete	Yes	+++	1172.09	1499.72	+21.8	37.67	53.83	+69.9	2153.68	3192.85	+32.5	72.51	102.73	+29.4
7^c^	7	None	–	No	+	183.88	819.66	(+77.6)	7.68	33.49	(+77.0)	269.96	790.27	+65.8	17.8	34.41	+48.3
8	12	None	–	Yes	++	134.49	765.48	+82.4	6.06	29.01	+79.1	361.46	5501.74	+93.4	14.39	99.97	+85.6
Mean values						631.24	999.34	+48.8 (p = 0.018)	21.27	38.02	+59.4 (p = 0.0016)	1004.8	2773.9	+52.8 (p = 0.017)	39.5	75.3	+48.6 (p = 0.007)

*TAM* tibial anterior muscle, *ATA* anterior tibial artery, *RBV* regional blood volume, *RBF* regional blood flow

^a^+: satisfactory; ++: good; +++: very good

^b^Amputation at 5 month

^c^Amputation at 7 month

## Discussion

CLI is a manifestation of PAD that includes patients with typical chronic ischemic pain at rest or patients with ischemic skin lesions, either ulcers or gangrene. The term CLI should only be used in relation to patients with chronic ischemic disease, defined as symptoms lasting more than 2 weeks. The diagnosis of CLI should be confirmed by ABI, toe systolic pressure or transcutaneous oxygen tension. Ischemic rest pain most commonly occurs below an ankle pressure of 50 mmHg or a toe pressure less than 30 mmHg [1].

The first large report on the use of bone marrow-derived mononuclear cells (BM-MNC) in limb ischemia was the therapeutic angiogenesis by cell transplantation (TACT) study by Tateishi-Yuyama et al. At 4 weeks, ankle-brachial index (ABI) was significantly improved in legs injected with BM-MNC and similar improvements were seen for transcutaneous oxygen pressure. They concluded that autologous implantation of BM-MNC could be safe and effective for achievement of therapeutic angiogenesis, because of the natural ability of marrow cells to supply endothelial progenitor cells and to secrete various angiogenic factors or cytokines [11]. Since then, several trials were published, both using BM-MNC, and mobilized peripheral blood mononuclear cells (PBMNC) with intra-arterial or intra muscular administration, well reviewed by Lawall et al. [12].

In their review, Lawall et al. sustain that the role of “EPCs” in human angiogenesis in the setting of peripheral vascular obstruction remains doubtful, and the translation of a truly “EPC” based endothelial repair into clinic practice has not been achieved so far. The well substantiated concept of arteriogenesis strengthens the importance of several different bone marrow cell types, however all sharing a monocytic phenotype. They migrate to the perivascular space of sprouting collaterals and induce collateral artery growth by the release of angiogenic growth factors. A growing body of evidence strongly suggests that these secreted molecules mediate a number of protective mechanisms including cell survival, neo-vascularization, remodeling, and proliferation. The regulatory system governing paracrine factor release appears to be complex and dependent on spatiotemporal parameters. [13].

Based on available data, cell therapies in PAD based on the application of whole BM-MNC or on whole stimulated PBMNCs are more successful than methods which use subfractionated cell preparations [12], e.g. CD 133+ [14] or highly purified CD 34+ cells from peripheral blood after granulocyte- colony stimulating factor (G-CSF) mobilisation only [15]. Nevertheless available data are very scarce about efficacy of a specific subset cells population versus the entire pool of mononuclear cells, because great majority of the previous and ongoing studies employ BM-MNC or PBMNCs, due to both lower costs and relative simplicity of the method. Except the case report of Canizo et al., the only previous study employing autologous selected CD 133+ cells is by Burt et al.: they treated nine patients with positive results in 7 [16].

Previous EPCs in CLI studies considered only data measured with ABI and TcPO_2_. As a matter of fact both ABI and TCpO2 have an intra and inter patient variability due to detection method, operator experience, vasodilatation state and emotional stress, that make standardization difficult. Moreover, in case of very low velocity flow, as in peripheral blood circulation in CLI, ABI detection variability increases [17]. CEUS offers a reliable method to measure peripheral blood flow, and is a valid alternative: it’s equally a non-invasive method, because the medium contrast hasn’t got any contraindication, except hypersensitivity, and the assessment method is not operator, but dedicated software related. It has only a minimal intra patient variability, when the detection method is accurate.

The aims of our study are (1) to demonstrate that a highly purified autologous stem cells population can induce neo-angiogenesis in a safe, feasible and effective way and (2) to assess neo-angiogenesis with a non-invasive method as most objective and reproducible as possible. In consideration of the end stage disease character of CLI, we didn’t considered ethical randomizing eligible patients. The possible advantages in studying a specific cells population in order to understand the mechanisms of neo-angiogenesis are to avoid the overlapping effects of entire MNC populations infused and as second step to better identify the cytokines pattern, derived from a single cell type, given the hypothesis of a paracrine mechanism.

Stem cells mobilization with G-CSF administration induce a high white blood cells count (WBCc) and a subsequent theoretical blood hyper viscosity. In patients with CLI blood hyper viscosity can be an issue. In our series the mean WBC count at the 4th day after mobilization was 48.1 × 10^6^/ml (range 26.6–77). These values are similar to these observed in healthy donors mobilized for allogeneic hematopoietic stem cell transplant. Even in the patient (ID = 7) with the highest WBCc (77.0 × 10^6^/ml) we didn’t observe any significant side effect related to blood hyper viscosity. However, we administered a prophylactic dose of low molecular weight heparin (3800/4000 UI/daily) considering the pro thrombotic risk related to G-CSF administration and patient immobilization. Remarkably also the CD34+ and CD133+ mobilization in these patients is comparable to healthy donors showing that their stem cell reservoir is not depleted. Stem cell collection was performed following our internal protocol, processing 2.5 blood volumes without any relevant side effects. On the whole we can argue that patients with compromised peripheral circulation can tolerate very well both mobilization and LKF. The wide range of CD133+ cell recovery after the ICS may be related to the different antigen expression on the stem cell surface. All the immunoselected CD133+ cell samples showed an high in vitro clonogenic potential (similarly to hematologic field) demonstrating the good quality of the product infused. Nevertheless the purity was always very high (≥90 %), except in one case (ID = 5), showing that every patient, but one, was treated with a single cell population. From this point of view, we obtained results comparable with the study of Burt et al. [16].

Altogether, 6 out of 8 patients (75 %) had clinical improvement, with ulcer healing, cessation of rest pain, increased walk pain free distance and above all, avoided amputation and maintained their improvement for a long period of time. Indeed they had the longest follow up so far in literature, 12 months with CEUS and at least 18 months from a clinical point of view. The graphics in Fig. 1 show the increase (in percentage) from baseline of RBV and RBF, both for ATM and ATA: mean percentage increase (continuous line) is always positive and statistically significant (see also Table 4 for p values). Clinical improvement is consistent with instrumental data showing an increased blood flow in the limb, reasonably related to an induced neo-angiogenesis. Nevertheless other important local factors may be involved both in initiating and maintaining angiogenesis, like resident cells and chemokines and cytokines environment. Data demonstrate that the resident progenitor cells could differentiate into a variety of cell types in response to different culture conditions. However, collective data were obtained mostly from in vitro culture assays and phenotypic marker studies. There are many unanswered questions concerning the mechanism of cell differentiation and the functional role of these cells in vascular repair and the pathogenesis of vascular disease [18].

Patient 3, despite the maximum values of cell dose infused (42.2 × 10^6^/limb kg) showed a complete lack of responsiveness both clinical, and instrumental (Table 4). Conversely, Patient 5 received the lowest dose of CD133+ cells with the lowest purity (Table 3): however she showed a satisfactory clinical response (ulcer healing, rest pain relief and increased pain free walk distance) and TAM-RBV/RBF increasing (+28.6 and +42.9, respectively). We can speculate that these opposite results are related not exclusively to the cell dose infused, but also to responsiveness of the resident stem cells. Indeed we didn’t find any statistical correlation between the number of infused CD133+ cells (10^6^ per limb kg) and the clinical and CEUS results.

Patient 7 showed apparently conflicting results: at 6 months TAM-RBV/RBF were increased around 77 % with a slightly improvement of clinical condition. Unfortunately the following month she developed a sudden worsening in the limb ischemia, with subsequent above the knee amputation. It’s crucial to emphasize that the patient had insulin dependent diabetes mellitus (IDDM) and a heavy smoking habit (around 40 cigarettes daily). We can suppose a negative role of IDDM and a precipitating role of the smoke habit during the transient neo-angiogenesis process, as assessed by CEUS values increasing. However impaired angiogenesis in diabetes has been already demonstrated both in animal models and in humans [19, 20].

In conclusion, our study shows interesting perspective and issues. Highly purified autologous CD133+ cells, routinely employed in transplant for oncohematologic diseases, can stimulate neo-angiogenesis, either directly or through a paracrine effect, as based on clinical (ulcer healing, rest pain cessation, increasing pain free walk distance and limb salvage) and CEUS data. Our study gives an instrumental demonstration of neo-angiogenesis. The limits of the study are the lack of randomization, which we judged unethical for this kind of end-stage disease, and the low number of patients, mainly due to the restrictive inclusion and exclusion criteria. Goals for future studies are to enrol a major number of patients including also less advanced stages of PAD, and to consider other powerful stem cell sources as cord blood derived or mesenchymal stem cells.

## Additional files

10.1186/s12967-015-0697-4 The procedure of implant through multiple intramuscular 1 ml injection of CD133+ cells suspension.
10.1186/s12967-015-0697-4 The complete healing of a deep and painful ischemic lesion with bone exposure in patient 1. Beside the corresponding change in RBV and RBF measurements.
