# Supplementary figures and images for: Autologous immuno magnetically selected CD133+ stem cells in the treatment of no-option critical limb ischemia: clinical and contrast enhanced ultrasound assessed results in eight patients
Source: J Transl Med. 2015 Nov 3;13:342. doi: 10.1186/s12967-015-0697-4 (PMC4630831; doi:10.1186/s12967-015-0697-4)

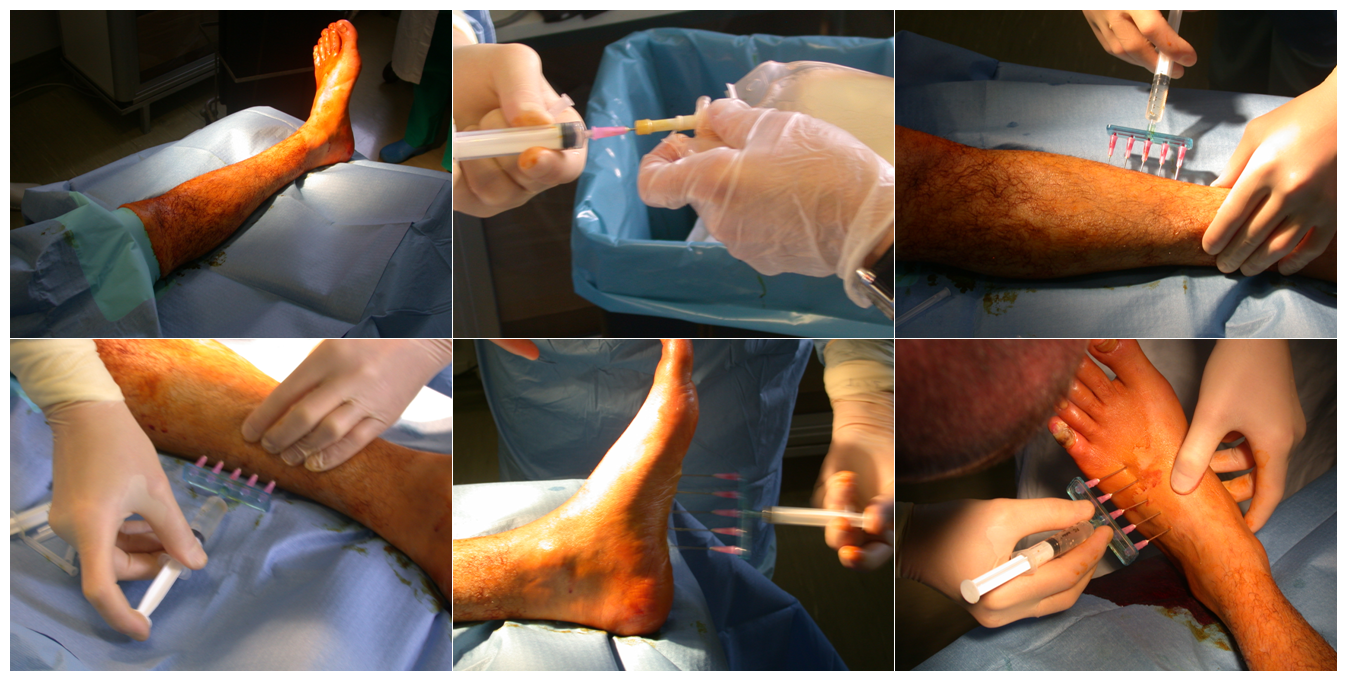

Supplement: Supplementary file 1 — 10.1186/s12967-015-0697-4 The procedure of implant through multiple intramuscular 1 ml injection of CD133+ cells suspension. [file 12967_2015_697_MOESM1_ESM.tiff]

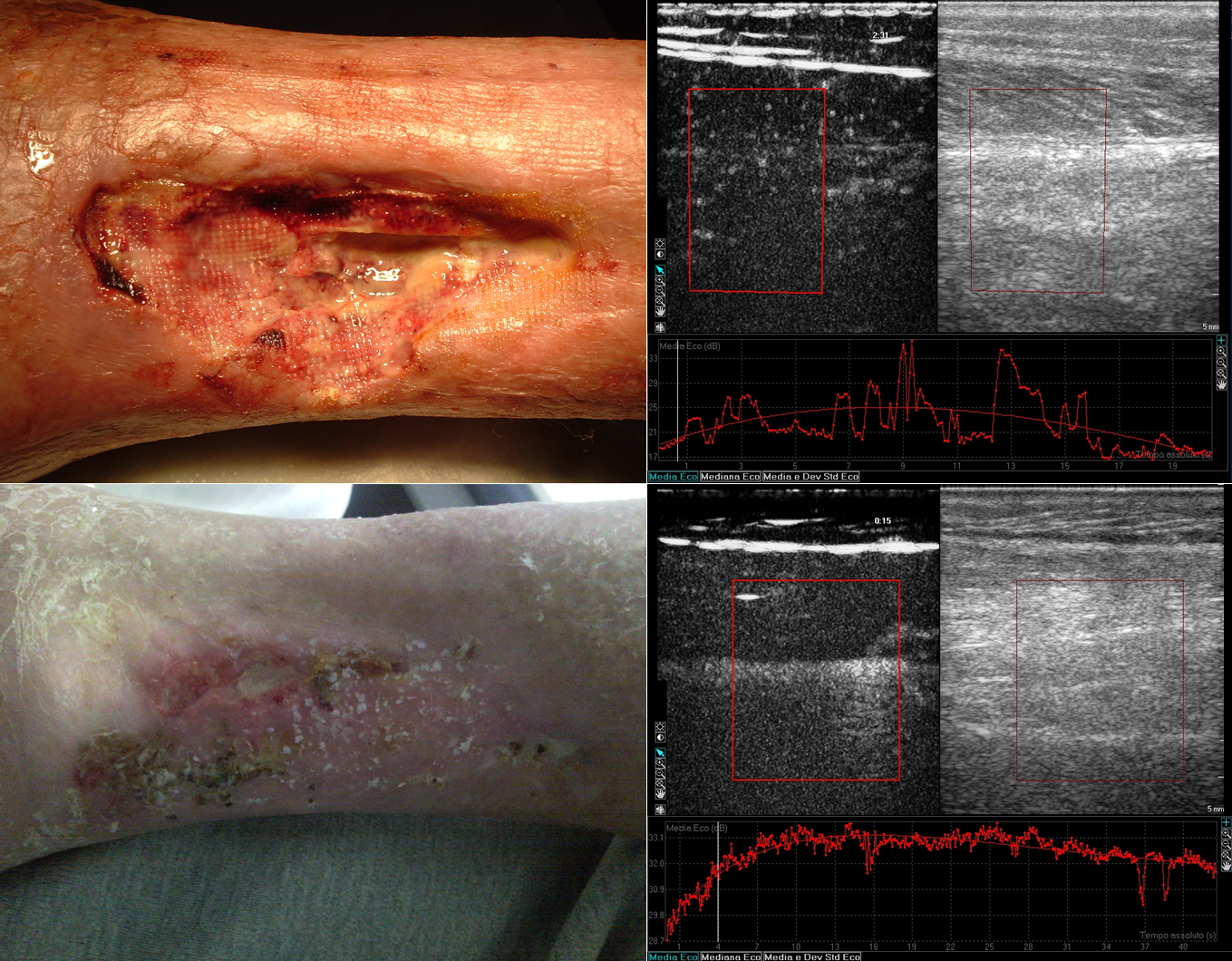

Supplement: Supplementary file 2 — 10.1186/s12967-015-0697-4 The complete healing of a deep and painful ischemic lesion with bone exposure in patient 1. Beside the corresponding change in RBV and RBF measurements. [file 12967_2015_697_MOESM2_ESM.tiff]
